# New tricks for p53 regulation – restraint by protein coding RNAs

**DOI:** 10.1186/s13578-015-0022-1

**Published:** 2015-06-12

**Authors:** Christine Blattner

**Affiliations:** ITG-Institute of Toxicology and Genetics, Karlsruher Institut für Technologie (KIT), PO Box 3640, 76021 Karlsruhe, Germany

**Keywords:** p53, DNA damage, Apoptosis, Embryonic stem cells

## Abstract

P53 is most well-known for its tumor suppressive function in differentiated cells. Its activities in embryonic stem cells (ESCs) are, however, less well understood. For many years it was thought that p53 is not active at all in ESCs and unable to elicit a DNA damage response in this cell type. In the last few years, it emerged that p53 may have some functions in ESCs. Nevertheless, it remained a mystery how its activity is controlled in ESCs. A recent report demonstrates that p53 activity is regulated by a novel RNA-containing negative feedback loop that promotes apoptosis specifically in ESCs. This study not only demonstrates unequivocally that p53 is active in ESCs, it further illustrates a novel mechanism of gene regulation–by protein coding RNAs.

## Text

Embryonic stem cells (ESCs) are pluripotent and can differentiate into many cell types. To avoid passing mutations to daughter cells, ESCs have great need to maintain their genome integrity. The tumor suppressor p53 plays proverbial roles in regulating the genomic integrity of ESCs. Several studies have established that p53 induces ESC differentiation in response to DNA damage [1, 2]. However, whether p53 promotes ESC apoptosis has not been widely studied since an early report suggested that p53 is inactive in ESCs [3]. Recent studies raised the possibility that p53 has a pro-apoptotic role in mouse ESCs [4, 5]. However, the molecular mechanism is still lacking. A recent report by Huang and his colleagues in *Cell Stem Cell* has shed light on the intricate pathway that regulates the pro-apoptotic function of p53 in ESCs [6].

To investigate the mechanism, Huang and his colleagues set out to test whether p53 induces apoptosis in mouse ESCs upon DNA damage and found that p53 is critical for DNA damage-induced apoptosis (DIA). Thus, the pro-apoptotic roles of p53 are conserved between stem cells and differentiated cells. They then used genomic approaches and identified a number of potential downstream targets that mediate p53’s pro-apoptotic function. Since little is known about the mechanism of gene repression by p53, the authors decided to focus on one transcript called *Apela* for further study that is repressed by p53 and highly expressed in ESCs. They found that *Apela* positively regulates p53-mediated DIA in mouse ESCs. Surprisingly, although *Apela* RNA has a putative coding region, this region is dispensable for the function of *Apela* in the DIA of mouse ESCs. Instead, *Apela* acts as a regulatory RNA. But as no direct interaction could be found for *Apela* and p53, how can this RNA regulate p53 function? To answer this question, the authors performed RNA-precipitation. One of the proteins that precipitated with *Apela* was the heterogenous nuclear ribonucleoprotein L (hnRNPL). This protein associates with p53 and inactivates the tumor suppressor protein by sequestering it from the mitochondrial department and by stimulating its degradation, the former but not the latter function is antagonized by *Apela*. In response to DNA damage, the relative interaction between hnRNPL and p53 is decreased, freeing p53 from its constraint and allowing its accumulation and initiation of apoptosis in ESCs. Huang and his colleagues speculate that *Apela* may facilitate a quick response of ESCs to DNA damage by skipping the translation step. As *Apela* is repressed by p53, p53, *Apela* and hnRNPL form a tri-element negative feedback loop in ESCs. Given the enrichment of *Apela* in ESCs, this negative feedback loop is most likely specific for the regulation of p53 activity in ESCs.

Because of its highly anti-proliferative activities, p53 needs to be kept in check and released from this constraint when its activity is needed. Therefore, several regulatory mechanisms have been implemented in cells that control p53 abundance and activity in stem cells as well as in differentiated cells including regulation of abundance and activity by posttranslational modifications [7], association with inhibitory proteins [7], regulation by micro-and other non-coding RNAs [8, 9] and now as shown in a very elegant way by Huang and his colleagues also by a protein coding RNA [6]. Whether this mode of control is a general means for gene regulation or whether it is restricted to the regulation of p53 activity in ESCs merits future investigations.
